# Co-Application of Biochar and *Arbuscular mycorrhizal* Fungi Improves Salinity Tolerance, Growth and Lipid Metabolism of Maize (*Zea mays* L.) in an Alkaline Soil

**DOI:** 10.3390/plants10112490

**Published:** 2021-11-17

**Authors:** Ndiaye Ibra Ndiate, Qudsia Saeed, Fasih Ullah Haider, Cai Liqun, Jackson Nkoh Nkoh, Adnan Mustafa

**Affiliations:** 1 College of Resources and Environmental Sciences, Gansu Agricultural University, Lanzhou 730070, China; ibra@st.gsau.edu.cn (N.I.N.); fasihullahhaider281@gmail.com (F.U.H.); 2 Gansu Provincial Key Laboratory of Arid Land Crop Science, Gansu Agricultural University, Lanzhou 730070, China; 3 College of Natural Resources and Environment, Northwest Agriculture and Forestry University, Xianyang 712100, China; syedaqudsia.saeed@yahoo.com; 4 Organization of African Academic Doctors, Off Kamiti Road, Nairobi 25305-00100, Kenya; nkohjackson@issas.ac.cn; 5 State Key Laboratory of Soil and Sustainable Agriculture, Institute of Soil Science, Chinese Academy of Sciences, P.O. Box 821, Nanjing 210008, China; 6 Biology Center CAS, SoWa RI, Na Sadkach 7, 370-05 České Budějovice, Czech Republic; adnanmustafa780@gmail.com

**Keywords:** biochar, alkaline soils, abiotic stress, *Arbuscular mycorrhizal* fungi, fatty acids, *Zea mays* L.

## Abstract

This study reports the mitigating strategy against salinity by exploring the potential effects of biochar (5%), *Arbuscular mycorrhizal* fungi (20 g/pot, AMF), and biochar + AMF on maize (*Zea mays* L.) plants grown under saline stress in a greenhouse. The maize was grown on alkaline soil and subjected to four different saline levels; 0, 50, 100, and 150 mM NaCl. After 90 d for 100 mM NaCl treatment, the plant’s height and fresh weight were reduced by 17.84% and 39.28%, respectively, compared to the control. When the saline-treated soil (100 mM NaCl) was amended with AMF, biochar, and biochar + AMF, the growth parameters were increased by 22.04%, 26.97%, 30.92% (height) and 24.79%, 62.36%, and 107.7% (fresh weight), respectively. Compared to the control and single AMF/biochar treatments, the combined application of biochar and AMF showed the most significant effect in improving maize growth under saline stress. The superior mitigating effect of biochar + AMF was attributed to its effective ability in (i) improving soil nutrient content, (ii) enhancing plant nutrient uptake, (iii) increasing the activities of antioxidant enzymes, and (iv improving the contents of palmitoleic acid (C16:1), oleic acid (C18:1), linoleic acid (C18:2), and linolenic acid (C18:3). Thus, our study shows that amending alkaline and saline soils with a combination of biochar-AMF can effectively mitigate abiotic stress and improve plant growth. Therefore, it can serve as a reference for managing salinity stress in agricultural soils.

## 1. Introduction

Biochar is an alkaline byproduct of the fast or slow pyrolysis of different biomass in a limited oxygen environment. Due to its high pH, biochar application to soils is mostly popular for the management of acidic soils [1,2]. Studies have shown that when applied to alkaline soils, biochar decreased the soil bulk density, cation exchange capacity, organic carbon content, nitrate retention, and bioavailable potassium [3,4]. The negative effect of biochar on the availability of phosphorus in alkaline soils was reported by Baigorri et al. [5]. The authors observed that the adsorption of phosphorous to Al/Fe-modified biochar decreased its bioavailability and partially explained the observed negative effects of pristine biochar applied to alkaline soils. Understanding the role of pristine (unmodified) biochar in alkaline soils has been extended to studying their interactions with traditional NPK fertilizers. Mete et al. [6] showed that when biochar was applied together with NPK fertilizer, then it significantly improved seed yield and total biomass in three genotypes of soybean in alkaline soils. These different studies reveal that under the right conditions, biochar can induce beneficial effects on crops grown on alkaline soils.

In soils having a large concentration of soluble salts (e.g., containing Na^+^ ions), plants suppress growth due to the negative effects of high saline concentration on osmotic balance, ion homeostasis, and oxidative stress due to reactive oxygen species (ROS) [7,8]. According to Foyer and Noctor [9], ROS can disrupt cellular functions and negatively affect nucleic acids, activities of oxidant proteins, and induce lipid peroxidation. Thus, researchers have suggested that the accumulation of ROS under salinity stress in plants is one of the major causes of reduced global agricultural productivity [10]. The study of Farhangi-Abriz and Torabian [10] showed that under increased saline stress, the activities of catalase (CAT), ascorbate peroxidase, peroxidase (POD), and superoxide dismutase (SOD) in the leaves and roots of common bean (*Phaseolus vulgaris* L. cv. Derakhshan) were significantly increased. According to the authors, amending the soils with biochar suppressed saline-induced oxidative stress and improved the growth of the bean plant.

The management of saline soils with appropriate rates of biochar has a mitigating effect on N leaching, enhances N retention, and reduces volatilization of ammonia [11]. Also, the application of biochar to alkaline soils has been shown to favor the colonization of ammonia-oxidizing microorganisms and inhibit N leaching [12]. Cui et al. [13] reported that co-application of biochar and effective microorganisms significantly inhibited salinization, improved soil fertility, increased soil nutrient content, enhanced enzymes activities, thereby improving the growth of *S. cannabin*. The inoculation of saline contaminated soils with *Arbuscular mycorrhizal* fungi (AMF) strain *Glomus mosseae* improved the growth of tomato (*Lycopersicon esculentum* L. cv. Zhongzha105) plants by improving root colonization, contents of chlorophyll, fruit fresh weight, fruit yield, and total plant growth [14]. Mycorrhizal colonization can be enhanced when AMF is co-applied with biochar to mitigate the adverse effects of drought-related stress on plant growth [15]. The few studies that have reported on the combined application of biochar and AMF suggest that biochar modifies the physicochemical properties of soils which improves AMF colonization [16]. Due to the limited number of studies on the co-application of biochar and AMF in alkaline-saline soils, the specific mechanisms for improving plant growth in these soils is not clear. Thus, this study was designed to study the growth of maize (*Zea mays* L.) in alkaline soils under saline induced stress and to evaluate the effects of the combined application of AMF and biochar on maize growth parameters. It was hypothesized that AMF and biochar would have additive effects on plant growth, lipid metabolism, and nutrient availability, meaning that combining the two treatments would result in greater plant growth than either treatment alone under salinity stress.

## 2. Results

### 2.1. Effect of AMF and Biochar on Fatty Acids Composition of Maize Leaves

Table 1 shows the contents of fatty acids in the leaves of 90 days old plant under saline-induced stress. The contents of myristic acid (C14:0), palmitic acid(C16:0), palmitoleic acid (C16:1), stearic acid (C18:0), oleic acid (C18:1), linoleic acid (C18:2), linolenic acid (C18:3), arachidic acid (C20:0), and behenic acid (C22:0) were affected differently for the different treatments. The contents of C14:0, C16:0, and C18:0 were not significantly altered for different treatments compared to S0. With increasing salinity, the content of unsaturated fatty acids (C16:1, C18:1, C18:2, and C18:3) was observed to decrease for control (S1, S2, S3), biochar (BS1, BS2, BS3), and AMF (AS1, AS2, AS3) treatments. Compared to S0, the contents of C16:1, C18:1, C18:2, C18:3 was significantly (*p* < 0.05) decreased by 44.02%, 33.49%, 25.99%, and 0.53% in S3 treatment, respectively. Nevertheless, the contents of C20:0 (52.82%) and C22:0 (87.77%) were increased significantly (*p* < 0.05) improved for the same treatment. Also, relative to S3 treatment, BS3 treatment positively impacted the contents of C16:1, C18:2, C20:0, and C22:0while negatively affecting C18:1 and C18:3 (Table 1). For AS3 treatment, the contents of C16:1, C18:1, C20:0, and C22:0 were significantly decreased while C18:2 and C18:3 were only slightly increased. The combined application of biochar and AMF (ABS3) demonstrated the most significant positive effect on the contents of all fatty compared to S0 while C22:0 was negatively affected relative to S3. Specifically, the contents of C16:1, C18:1, C18:2, C18:3 and C20:0 were increased by 161.36%, 101.3%, 65.07%, 12.17%, 18.23% relative to S3 or 46.31%, 33.89%, 22.17%, 11.58%, 88.31%, and 56.46% relative to S0.

### 2.2. Influence of AMF and Biochar on Soil Nutrient Content

**Figure 1:**
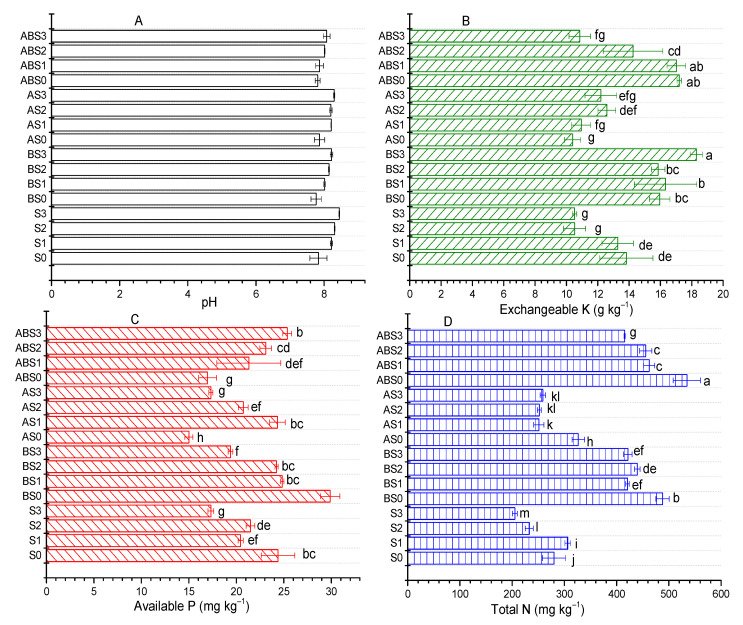
Influence of *Arbuscular mycorrhizal* fungi (AMF) and biochar on (**A**) soil pH, (**B**) exchangeable K, (**C**) available P, and (**D**) total N after harvesting. Data present the mean ± standard deviation of three replicates and mean values followed by different letters are significantly different at *p* < 0.05 according to Duncan multiple range test. S0, S1, S2, and S3 (0, 50, 100, and 150 Mm NaCl treatment, respectively); BS0, BS1, BS2 and BS3 (biochar + 0, 50, 100, and 150 Mm NaCl treatment, respectively); AS0, AS1, AS2 and AS3 (AMF + 0, 50, 100, and 150 Mm NaCl treatment, respectively); ABS0, ABS1, ABS2 and ABS3 (biochar + AMF + 0, 50, 100, and 150 Mm NaCl treatment, respectively).

The following acronyms will be used as defined in the materials and method section (Table 2): S0, S1, S2 and S3 (0, 50, 100, and 150 Mm NaCl treatment, respectively); BS0, BS1, BS2 and BS3 (biochar + 0, 50, 100, and 150 Mm NaCl treatment, respectively); AS0, AS1, AS2 and AS3 (AMF + 0, 50, 100, and 150 Mm NaCl treatment, respectively); ABS0, ABS1, ABS2 and ABS3 (biochar + AMF + 0, 50, 100, and 150 Mm NaCl treatment, respectively). Figure 1 gives a summary of the selected physicochemical properties of the soil after the growth of maize. After maize growth, the soil pH was decreased in the S0, S1, BS0, AS0, ABS0, ABS1 treatments by 0.42, 0.03, 0.48, 0.38, 0.44, 0.38 units while S2, S3, and A3 had an increase of 0.06, 0.20, and 0.05 units compared to the original soil (pH 8.25), respectively (Figure 1A). Compared to the S0 treatment, only the BS0 and ABS0 treatments experienced a slight decrease in pH. For different treatments, the content of potassium was either increased or decreased with an increment in the concentration of NaCl (Figure 1B). For instance, under no amendment, the content of K was decreased by 3.83%, 23.76%, and 23.8% when the concentration of NaCl was 50 (S1), 100 (S2) and 150 mM (S3), respectively. This decrement was also observed for AS0, AS1, AS2, AS3, and ABS3 with values lowered by 24.68%, 20.72%, 8.82%, 11.64%, and 21.37%, respectively. This observation shows that increase in the concentration of Na^+^ ions has a negative impact on available K^+^ ions needed for plant growth, which has been sighted as a major concern for saline soils [17]. Nevertheless, amending the alkaline soil with biochar (BS0, BS1, BS2, BS3) and biochar + AMF (ABS0, ABS1, and ABS2) significantly (*p* < 0.05) improved the content of K; with the largest increment recorded for BS3 (32.49%), ABS0 (24.62%), ABS1 and (23.31%) relative to S0. When compared with the saline-treated control (S1, S2, S3), biochar (BS1, BS2, BS3), AMF (A1, A3, except A1), and biochar + AMF (ABS1, ABS2, ABS3) treatments significantly (*p* < 0.05) improved K in soil. Thus, biochar amendment with/without AMF can effectively mitigate K^+^ ion loss in high pH saline soils. Phosphorus and nitrogen are important nutrient requirements for plant growth which can become deficient when the soil health and fertility are threatened. For the control (S0, S1, S2, and S3) and AMF (AS0, AS1, AS2, and AS3) treatments, the content of P was decreased as the concentration of NaCl was increased (Figure 1C). Relative to the S0 treatment, the content of P was increased by 22.58% for B_0_ but was decreased by 38.45% and 30.41% for A_0_ and AB_0_ treatments, respectively. Also, relative to the saline (S1, S2, S3) treatments, biochar (BS1, BS2, BS3), AMF (AS1, AS2, AS3), and biochar + AMF (ABS1, ABS2, ABS3) amendments showed contrasting effects on available P. For instance, the available P was increased by 21.43%, 12.78%, 11.97%, 4.23%, 7.43%, 46.43%, 18.94% for BS1, BS2, BS3, ABS1, ABS2, ABS3, and AS1, respectively, but decreased for AS2 and AS3 treatments. From this result, it is evident that salinity negatively impacts available P and the individual or co-application of biochar with AMF can mitigate this negative effect.

From Figure 1, it can be observed that the content of N significantly decreases in the control treatment as the saline content was increased from 0–150 mM; except for S1 treatment (Figure 1D). For instance, compared to the S0 treatment, N was increased by 26.4 mg kg^−1^ and decreased by 47.3 and 74.9 mg kg^−1^ when the soil was treated with 50 (S1), 100 (S2), and 150 Mm (S3) of NaCl, respectively. The application of biochar alone (BS0, BS1, BS2, BS3) and in combination with AMF (ABS0, ABS1, ABS2, ABS3) significantly improved the content of N under increasing salt stress. For no salt treatments (BS0, AS0, and ABS0), N content was improved by up to 74.25%, 16.61%, and 90.89% for BS0, AS0, and ABS0 amendments relative to S0 treatment, respectively. Under increasing salt stress, N content was improved progressively when biochar and/or AMF were applied. Specifically, for BS1, BS2, and BS3 treatments, the amount of N (relative to S1, S2, and S3) increased by 37.3%, 88.91%, and 105.6% (or 50.25, 57, and 50.57% relative to S0), respectively. Similarly, the recorded increment in the content of N for ABS1, ABS2, and ABS3 was 50.88%, 95.79%, and 102.44% (or 65.11, 62.71, and 48.29% relative to S0), respectively. Additionally, applying AMF alone did not mitigate N loss (−18.05%) when 50 mM NaCl was added but showed a positive effect at saline concentrations of 100 (8.25%) and 150 mM (26.08%). Thus, amending alkaline soils with biochar, biochar + AMF, and AMF alone can mitigate the negative effect of Na^+^ ions on N content, with biochar and biochar + AMF exhibiting the most significant effects at 100 and 150 mM of Na^+^ ions.

### 2.3. The Effects of Salinity, Biochar, and AMF on Plant Growth Parameters

**Figure 2:**
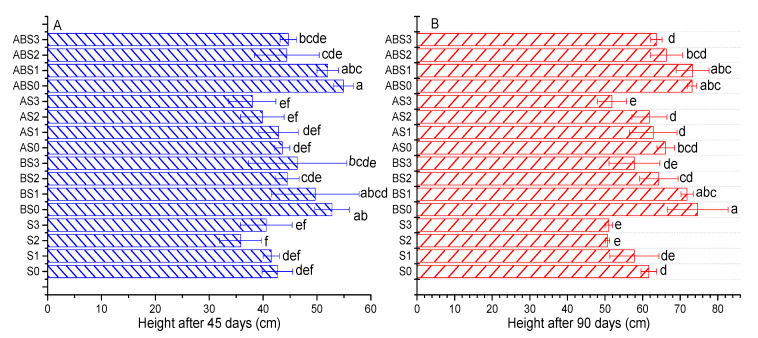
Impact of AMF and biochar on the height of corn plants under different levels of salinity after 45 (**A**) and 90 (**B**) days of growth. Data present the mean ± standard deviation of three replicates and mean values followed by different letters are significantly different at *p* < 0.05 according to Duncan multiple range test. The significance of acronyms is the same as in Figure 1.

**Figure 3:**
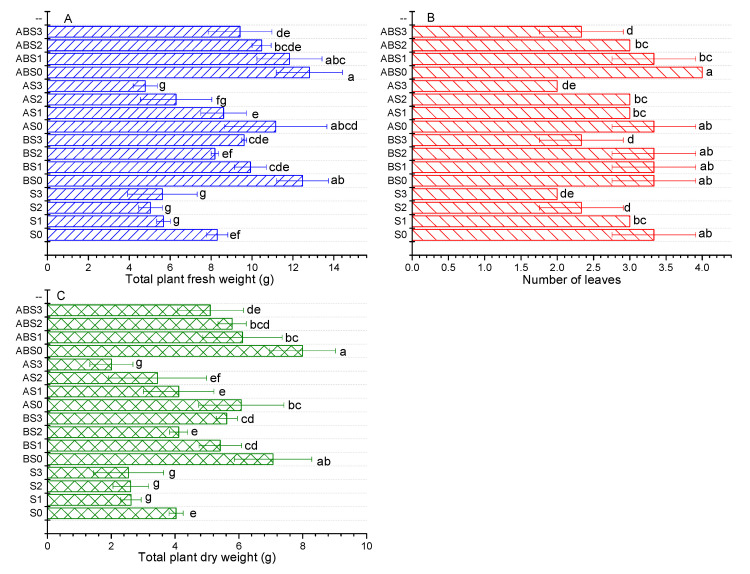
Impact of AMF and biochar on plant fresh weight (**A**), number of healthy leaves (**B**), and dry weight (**C**) under different concentrations of salinity. Data present the mean ± standard deviation of three replicates and mean values followed by different letters are significantly different at *p* < 0.05 according to Duncan multiple range test. The significance of acronyms is the same as in Figure 1.

Plant growth parameters were evaluated based on the maize height after 45 and 90 days of growth, total fresh weight, and the total number of leaves after 90 days (Figure 2 and Figure 3). The plant height after 45 days of growth was 2.73%, 16.02%, and 4.92% shorter compared to S0 when the saline concentration was 50 (S1), 100 (S2), and 150 mM (S3), respectively (Figure 2A). After 90 days of growth for the same treatments (Figure 2B), the plant height was 6.22%, 17.84%, and 17.3% shorter, respectively. When the soil was amended with biochar and/or AMF without NaCl, the plant height was increased by 23.67%, 28.67%, 2.11% after 45 days and by 21.08%, 18.65%, 7.3% after 90 days for BS0, ABS0, and AS0 compared to S0, respectively. Under increasing saline stress, biochar enhanced plant growth by 19.68% (BS1), 24.19% (BS2), 14.38% (BS3) after 45 days and by 24.21% (BS1), 26.97% (BS2), 13.4% (BS3) after 90 days when compared to S1, S2, and S3, respectively. This significant (*p* < 0.05) increment in the plant height was also recorded when the soil was amended with biochar + AMF (ABS1, ABS2, and ABS3). Comparatively, the biochar treatments demonstrated a significant effect in improving the plant’s height compared to single AMF treatment; with biochar + AMF being a better option. 

Figure 3 shows the difference in the total plant fresh weight (Figure 3A), the number of leaves (Figure 3B), and the total plant dry weight (Figure 3C). As shown, increasing the concentration of NaCl negatively affects the total plant fresh weight (Figure 3A). Increasing the NaCl concentration by 50 (S1), 100 (S2), and 150 mM (S3) significantly decreased the plant’s fresh weight by 31.62%, 39.28%, and 32.28% relative to S0, respectively. When no salt was applied to the soil, biochar and AMF amendments improved the plant’s fresh weight by 50.21%, 54.28%, and 34.4% for BS0, ABS0, and AS0, respectively. Compared to the saline treatments (S1, S2, and S3), biochar amendments improved the plant’s fresh weight by 74.84%, 62.36%, 71.14% for BS1, BS2, BS3 and 108.5%, 107.7%, 67.48% for ABS1, ABS2, and ABS3, respectively. For the treatments containing only AMF, the plant fresh weight was increased for AS1 (51.49%) and AS2 (24.76%) but was decreased as the salt content was increased to 150 mM for AS3 (−14.91%). Compared to the S0 treatment, all the treatments with biochar induced a significant improvement in plant fresh weight and demonstrates the individual ability of biochar or when combined with AMF to alleviate saline-related stress on plant growth.

The mean number of healthy leaves decreased with increasing salt stress for the control treatment (S1, S2, S3) (Figure 3B). Amending the soil with biochar (BS0, BS1, BS3) or combined with AMF (ABS0, ABS1, ABS2) or with AMF alone (AS0, AS1, AS2) mitigated the adverse effect of increasing saline concentration on the number of healthy leaves. Nevertheless, at a higher saline concentration (150 mM), the different amendments had fewer leaves when compared to S0 but higher when compared to the S3 treatment.

The total plant dry weight (Figure 3C) was negatively affected by increasing NaCl concentration. This observation corroborates the negative impact of saline stress on plant water content observed in Figure 3A. The plant dry weight was 4.03 g for S0 treatment but was decreased by 35.06%, 35.21%, and 36.95% in the S1, S2, and S3 treatments, respectively. The different amendments demonstrated positive impacts on the plant dry weight, both in the absence and presence of saline stress. For instance, biochar (BS0), AMF (AS0), and biochar + AMF (ABS0) increased the plant dry weight by up to 75.38%, 50.74%, and 90.31% when compared to S0, respectively. Under increased NaCl concentration, biochar, AMF (except AS3), and biochar + AMF treatments significantly improved the plant dry weight. For example, at the highest saline concentration and when compared to S0, the plant dry weight was 76.4% (BS3) and 63.6% (ABS3) larger than the corresponding untreated S3 sample.

### 2.4. The Effects of Salinity, Biochar, and AMF on the Nutrient Uptake Ability of the Maize Plant during Growth

**Figure 4:**
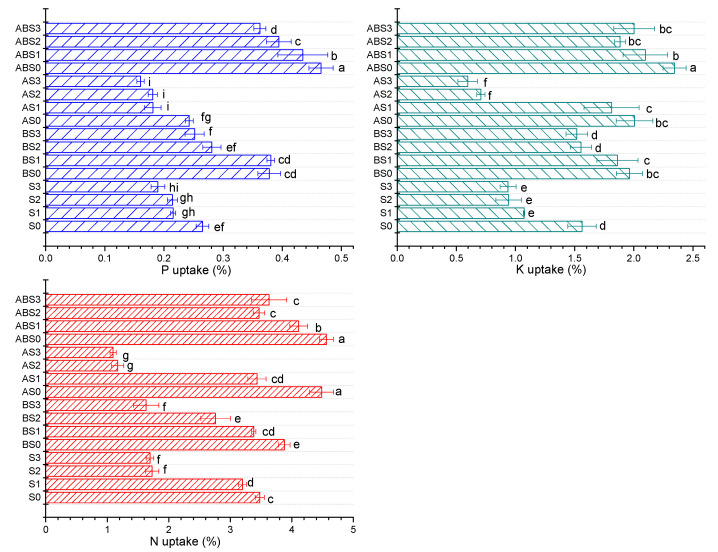
Effect of biochar and/or AMF on percentage of N, P, and K uptake by the maize plant under salinity stress. Data present the mean ± standard deviation of three replicates and mean values followed by different letters are significantly different at *p* < 0.05 according to Duncan multiple range test. The significance of acronyms is the same as in Figure 1.

Figure 4 shows the nutrient uptake ability of maize plants under different saline concentrations before and after amending the soil with biochar and/or AMF. It can be observed that increasing the saline concentration from 0 to 150 mM (S0 to S3) induced a negative effect on the nutrient uptake ability of the plant. Specifically, %N was decreased from 3.481 to 1.694, %P from 0.265 to 0.189, and %K from 1.562 to 0.937. This corroborates the observed negative effect of increasing salt stress on maize growth observed above (Figure 2 and Figure 3). Under no salt stress, the biochar and AMF treatments (AS0, ABS0, and BS0) enhanced the nutrient uptake ability of the plant compared to S0 treatment. Under increasing saline stress, all treatments containing biochar exhibited a superior effect on promoting nutrient uptake compared to single AMF treatments, with a combined application of biochar + AMF showing the best effect relative to S0 and saline controls (S1, S2, and S3). From this result, it can be inferred that the application of biochar and/or AMF to alkaline and saline soils can enhance plant growth by suppressing salt-related stress thereby improving soil fertility, improving nutrient availability, and promoting the uptake of essential nutrients required for growth.

### 2.5. Influence of AMF and Biochar on Photosynthetic Pigments in Plant

**Figure 5:**
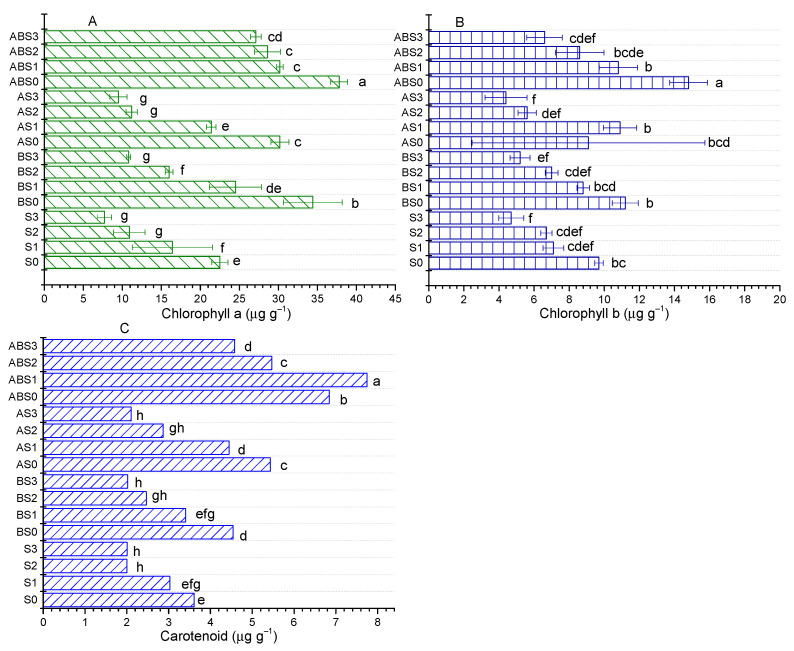
Effect of biochar and AMF on photosynthetic pigments: (**A**) Chlorophyll a; (**B**) Chlorophyll b; (**C**) Carotenoid. Data present the mean ± standard deviation of three replicates and mean values followed by different letters are significantly different at *p* < 0.05 according to Duncan multiple range test. The significance of acronyms is the same as in Figure 1.

The effect of increasing saline concentration and the mitigating effects of different amendments on photosynthetic pigments were evaluated and the results are shown in Figure 5. Compared to S0, the content of chlorophyll a was decreased by 27.11%, 51.56%, and 65.78% (S_3_) as the salt content was increased in S1, S2, and S3, respectively (Figure 5A). Similarly, the contents of chlorophyll b were decreased by 26.8%, 30.93%, and 51.55% (Figure 5B) while carotenoid was decreased by 16.08%, 44.48%, and 44.41% (Figure 5C) in S1, S2, and S3, respectively. From this result, it is evident that under increasing salt stress, plant growth becomes inhibited as the required nutrients are made less available which results in a reduced photosynthetic rate. 

Amending the alkaline soil with biochar alone or combined with AMF significantly improved the plant’s photosynthetic ability as chlorophyll a, chlorophyll b, and carotenoids were significantly (*p* < 0.05) improved. The contents of chlorophyll a, chlorophyll b, and carotenoids were increased by 52.89%, 15.46%, 25.78% for B_0_ and 68%, 52.58%, 89.46% for AB_0_ compared to S0, respectively. Similarly, the A_0_ treatment improved the contents of chlorophyll a and carotenoids by 34.22% and 50.34%, respectively, while decreasing that of chlorophyll b by 6.19%. Under increasing saline stress, all amendments containing biochar showed positive effects on improving the photosynthetic ability of the maize plant while the single AMF treatment demonstrated negative effects on chlorophyll b at 100 (AS2) and 150 mM NaCl (AS3). Of all treatments, the combined application of biochar + AMF induced the most significant effect on improving the contents of photosynthetic pigments under salt stress. Relative to the S0 treatment, the content of chlorophyll a was 61.33%, 78.67%, 86.22%; chlorophyll b was 38.14%, 19.59%, 19.59%; and carotenoid was 130.6%, 95.89%, 71.18% higher in ABS1, ABS2, ABS3 treatments than in the S1, S2, S3 treatments, respectively. This observation is in agreement with the superior ability of biochar + AMF treatments in significantly enhancing nutrient uptake by the plant (Figure 4) under increased saline stress relative to other treatments. Therefore, amending alkaline and saline soils with AMF, biochar, or biochar + AMF can play an important role in mitigating stress-related adverse effects in these soils that inhibit plant growth.

### 2.6. Impact of AMF and Biochar on Antioxidant Enzyme Activities

**Figure 6:**
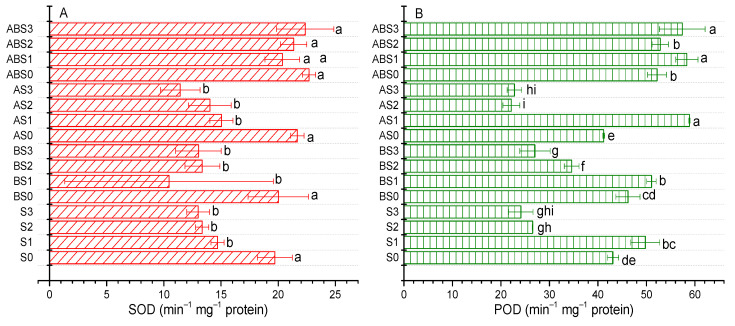
Impact of AMF and biochar on the activity of SOD (**A**) and POD (**B**) in maize under different levels of salinity. Data present the mean ± standard deviation of three replicates and mean values followed by different letters are significantly different at *p* < 0.05 according to Duncan multiple range test. The significance of acronyms is the same as in Figure 1.

The activities of SOD and POD in the leaves of the maize plant were significantly (*p* < 0.05) affected after 90 days of growth under different saline conditions (Figure 6). Compared to S0, increasing the saline concentration reduced the activity of SOD by 25.41%, 32.26%, and 34.04% in S1, S2, and S3 treatments, respectively (Figure 6A). For similar treatments, the content of POD was increased by 15.41% for S1butdecreased by 38.44% for S2 and 44.09% for S3. After amending the soil with biochar (BS0), AMF (AS0), and biochar + AMF (ABS0), the activity of SOD was increased by 1.55%, 10.01%, and 15.18%, respectively. Also, while the BS0 (7.14%) and ABS0 (21.04%) treatments enhanced the activity of POD, the AS0 treatment induced a 4.41% decrease in its activity compared to S0.

Under different saline conditions, the amendments showed diverse effects on the activities of SOD and POD. Relative to S0 treatment, the content of SOD in biochar amendments was lower by 21.52% (BS1) or insignificantly higher by 0.08% (BS2) and 0.17% (BS3) when compared to S1, S2, and S3, respectively. Similarly, an insignificant increase was observed for AS1 (1.69%) and AS2 (3.42%) and a decrease for AS3 (7.91%). The application of biochar mitigated the adverse effect of saline stress on the activity of POD (BS1, BS2, BS3) while AMF only had a positive effect at a lower saline concentration (AS1). Of all treatments, the combined application of biochar and AMF showed the most significant (*p* < 0.05) effect on the activities of SOD and POD under saline stress. Compared to S0, the activity of SOD was 28.74%, 40.63%, and 47.5% higher for ABS1, ABS2, ABS3 than for S1, S2, S3, respectively. Similarly, the activity of POD was19.86%, 61.03%, and 77.21% higher in ABS1, ABS2, ABS3 treatments than for S1, S2, S3, respectively. This result shows that biochar + AMF had the most significant effect on the activities of SOD and POD under increasing saline stress, making this combination the best for improving antioxidant activities in alkaline and saline soils.

## 3. Discussion

The application of biochar to alkaline soils have demonstrated both positive and negative effects on the availability of nutrients such as K, P, and N. Chen et al. [18] showed that biochar nanoparticles can reduce P retention in alkaline soils by up to 23% and 18% and the authors associated this effect to increased leaching of P associated to Fe/Al oxides in soils. Contrarily, Cui et al. [13] observed that the co-application of biochar and effective microorganisms enhanced the growth of *Sesbania cannabina* in coastal saline-alkali soil. Our study shows that the application of biochar without/without *Arbuscular mycorrhizal* fungi (AMF) (*Glomus mosseae*) had a positive effect on the available nutrients (N, K, and P) in alkaline soil (Figure 1). Although the overall effect of biochar and/or AMF on soil pH was not significant, a significant increase was observed in the nutrient uptake ability of maize plants (Figure 4) and the measured growth parameters (Figure 2 and Figure 3).

Salt-affected soils are prone to nutrient deficiency and excess Na^+^ ions in soils affect the availability of plant nutrients either by direct competition or indirectly by increasing the osmotic pressure of the soil solution and retarding mass uptake of nutrients by the roots [19,20]. For saline soils, nutrients such as N, P, and K are in low quantities mostly due to low levels and rapid loss of organic matter [21]. This observation agrees with our results (Figure 1) given that the contents of N, P, and K were respectively reduced by 26.75%, 28.94%, and 23.8% when the NaCl concentration was increased to 150 mM for the control (S3). Under similar conditions, the individual application of biochar and combined application with AMF demonstrated the most significant effect in alleviating the adverse effects of saline stress on nutrient availability. Specifically, when compared to S0, the contents of N, P, and K were higher by 77.32%, 8.51%, and 56.3% in BS3 compared to S3 treatment, while in biochar + AMF (ABS3) treatment, it was 75.04%, 32.99%, 2.44% higher, respectively (Figure 1). 

Nutrient deficiency and low use efficiency have negative impacts on the photosynthetic potentials of plants and their subsequent resistance to stress [22]. When plants are under stress, they may suffer chlorophyll degradation which results in a reduction of the plant’s photosynthetic ability [23]. From Figure 5, it can be observed that increasing salinity reduced the plant’s photosynthetic ability. The saline-related stress significantly reduced the contents of photosynthetic pigments, with the reduction increasing with NaCl concentrations and most severe for chlorophylls a and b. Amending the soils with biochar and/or AMF alleviated the negative effects of increasing salinity on maize photosynthetic ability, with the biochar treatments being more effective than single AMF treatments. The colonization of plant roots by AMF has been reported to be beneficial for plant growth in low pH soils, and this effect was significantly improved when AMF was co-applied with biochar [15]. Thus, biochar application to soils played a modifying role in improving the physicochemical characteristics for effective colonization by AMF and it can be inferred that by improving soil fertility and nutrient uptake, biochar and/or AMF provided a conducive environment for plant growth.

The secretion of antioxidant enzymes is one key mechanism by which plants mitigate the adverse effects of biotic and abiotic stress [24,25]. By scavenging ROS, antioxidants can mitigate the adverse effects of oxidative stress induced by high saline concentrations thereby promoting plant growth [14]. As observed by Farhangi-Abriz and Torabian [10], an increase in the concentration of NaCl causes an increase in antioxidant enzymes such as CAT, SOD, and POD in the leaves and roots of bean seedlings. In our study, increasing the saline concentration decreased the contents of both SOD and POD (Figure 6) while co-application with biochar and/or AMF showed diverse effects. Nevertheless, applying biochar alone or combined with AMF mostly demonstrated an enhancing effect on the activities of SOD and POD; with the combined application of biochar + AMF showing the most significant (*p* < 0.05) increase. Our result may differ from that of Farhangi-Abriz and Torabian [10] primarily due to the difference in the plant types and growth stages considered.

The ability of plants to alter the contents of unsaturated fatty acids is an important mechanism through which they adapt to stress. Through the activities of fatty acid desaturases, plants modify their membranes to provide a suitable environment for the functioning of photosynthetic proteins [26]. Oleic acid (18:1) has been reported to be critical for resistance against pathogens [27] while linolenic acid (C18:3) is an important stress signal [28]. The importance of C18:1 and C18:2 levels have been documented in several studies and it was reported that they play important roles in the regulation of fungal development, seed colonization, and mycotoxin production by *Aspergillus* spp. [29,30]. When plants are subjected to saline stress, the content of C18:3 may decrease to indicate damages caused by the stress [26]. Under drought stress, *Brassica napus* leaves showed decreased levels of C18:3 and C18:2 [31]. Pál et al. [32] studied the effect of cadmium contamination on the content of membrane lipids of maize plants. They found that while the content of C16:0 in the leaves decreased that of C18:2 and C18:3 increased with increasing Cd levels. Also, they observed that for the roots, the levels of C18:0 and C18:1 decreased while those of C18:2 and C18:3 increased. We observed that saturated fatty acids (C14:0, C16:0, and C18:0) that play an important role in controlling the hydrophobicity of membrane proteins [33] were not significantly altered under saline stress and in the presence of biochar. Nevertheless, the levels of saturated acids with longer chains (C20:0 and C22:0) were significantly improved under increasing saline stress and in the presence of biochar. Conversely, increasing salinity negatively affected unsaturated fatty acid concentrations (C16:1, C18:1, C18:2) while the application of biochar demonstrated a mitigating effect and increased the levels of unsaturated fatty acids. The combined application of biochar and AMF showed the most significant effect in increasing the concentrations of unsaturated fatty acids. Thus, by improving the levels of membrane unsaturation, the co-application of biochar and AMF improved the growth of maize in alkaline soil under increasing saline stress. Therefore, we infer that under conditions of stress, plants become better adapted to handle different levels of stress by adjusting the fatty acid unsaturation levels. Also, soil amendments that can improve the content of fatty acid unsaturation may enhance plants’ adaptability to stress.

## 4. Materials and Methods

### 4.1. Experimental Materials

The agricultural soil used in this study was a loess soil collected from Lanzhou, China, with a bulk density of 1.25 g cm^−3^ and sampled from the surface 0–20 cm. The soil was air-dried and sieved with a 2 mm mesh sieve. On average, the annual rainfall of the study site was 415 mm and the annual temperature was about 6.2 °C. For soil characterization, five soil samples were collected and properly mixed to make a representative composite soil sample that had TN content of 1.74 ± 0.01 g kg^−1^, available phosphorus 25.1 ± 0.19 mg kg^−1^, soil organic carbon of 9.08 ± 0.09 g kg^−1^, and soil pH (in solution) 8.25 ± 0.11. Soil pH was determined using a METTLER TOLEDO Desktop pH meter after the soil sample was equilibrated in distilled water (1:5). Soil organic carbon was estimated by the Wet Oxidation method [34]. TN and phosphorus were determined by the Kjeldahl method [35] and Olsen method [36], respectively. The exchangeable K was evaluated by the Ammonium Acetate extraction method [37]. The soil test was performed in the laboratory of the College of Resources and Environmental Sciences at Gansu Agricultural University, Lanzhou, China. For this study, a pot experiment was conducted in greenhouse conditions (air temperature 30 °C; relative humidity 50%) from July to October 2020 at the Dingxi experimental station of Gansu Academy of Agricultural Sciences (Tangjiao Town, Dingxi city, 35°350 N, 104°360 E, 1970 m a.s.l), Gansu, China.

The biochar was prepared from corn straws collected from a cornfield in Tangjiao Town, Dingxi city, and transported to the College of Resources and Environmental Sciences of Gansu Agricultural University, Lanzhou, China. The straws were cut into 10–15 cm pieces and pyrolyzed at a temperature ranging from 350 to 500 °C for 1 h under an oxygen-deficient condition in an Oven-Electric Furnace (Heraeus MR 170, Meinerzhagen, Germany). After pyrolysis and cooling, the biochar produced was ground to pass a 250 µm mesh sieve and characterized. Total Organic Carbon (TOC) of biochar samples was measured after oxidization with potassium dichromate following Nelson and Sommers [38]. The total K contents were determined with a flame photometer (Jenway Flame Photometer, Bibby Scientific Ltd-Stone-Staffs-St15 0SA–UK.), P was estimated by a spectrophotometer as described by Sparks [39], and TN was determined by the micro-Kjeldahl method [40]. The %TN, %P, %K, and %TOC of biochar were 0.43 ± 0.01, 0.17 ± 0.004, 0.46 ± 0.003, and 41.2 ± 0.41, respectively. The seeds of corn (*Zea mays* L.) were collected from Gansu Provincial Key Laboratory of Arid Land Crop Science and kept at 4 °C for 24 h and later washed with running distilled water for 30 min before planting. Round plastic pots (21 cm in diameter * 16 cm in height) were used in this experiment. At the harvest period, the plant nutrient uptake (N, P, and K) were estimated following Hashem et al. [15]. To determine the contents of N and K in maize shoot, dried and ground samples were digested with H_2_SO_4_-H_2_O_2_ at 260–270 °C. N contents were determined by an Auto-analyzer 3 digital colorimeter (AA3, Bran + Luebbe, Hamburg, Germany) and K contents were estimated using Flame Photometry (FP6400, Shanghai Precision Scientific Instrument, Shanghai, China). The phosphorus was extracted by nitric-perchloric acid digestion and measured using the Vanado-molybdophosphoric colorimetric method. Standard curve of each mineral (10–100 µg^−1^ mL) used as reference.

### 4.2. Arbuscular Mycorrhizal Fungi (AMF) Inoculum

The Arbuscular mycorrhizal fungi (AMF) strain used was a single genus of *Glomus mosseae* provided by the Gansu Provincial Key Laboratory of Arid Land Crop Science, Lanzhou, China. It was multiplied with *Zea mays* (L.) as the host plant for 4 months in sterilized soil in the greenhouse of the College of Resources and Environmental Sciences of Gansu Agricultural University, Lanzhou, China. The methods previously described by Gerdemann and Nicolson [41] and Giovannetti and Mosse [42] were used to determine AMF inoculum characteristics. Root mycorrhizal colonization, soil spores content, arbuscules, and extraradical hyphae in the roots samples were observed accordingly. The AMF inoculum consisted of mycorrhizal roots (80% root mycorrhizal colonization), soil containing spores (50–80 per 10 g inoculum), and extraradical hyphae (2.5 m per 1 g soil) mixed with soil.

### 4.3. Experimental Treatments and Design

The greenhouse pot experiment was conducted in a completely randomized design with five replications per treatment and increasing salt stress. In total, there were 16 treatments as mentioned in Table 2 The NaCl concentration in the pots was gradually increased from 50 to 150 mM at a rate of 50 mM per 24 h. Frequent irrigations (thrice a week) with saline solution permitted the various salt concentrations in the pots to be maintained at a constant level. There was a total of 80 pots in the present greenhouse experiment. The biochar (5%) and/or AMF (20 g) were applied and thoroughly mixed with soil in each treated pot. This was followed by the sowing of eight imbibed seeds in each pot. After germination, the seedlings were thinned to four per pot.

### 4.4. Measurement of Plant Growth Parameters

The maize plant height was measured just before the final harvest. The height was measured by holding a measuring tape close to the stem of the plant. Plant height was recorded from the ground level to the base of the highest fully expanded leaf. The plant fresh weight (FW) was also determined by weighing the different parts of the harvested plant. The pots were cut away on two sides to permit careful root separation from the soil. For each treatment, plants were removed from the soil and washed with distilled water. After adsorbing residual water using tissue paper, an electronic balance was used to measure plant shoot and roots FW.

### 4.5. Lipid Extraction and Analysis

3 g 12-week-old maize leaves were harvested from plants grown on saline soils and transferred into 1.5 mL polypropylene reaction tubes. Fresh leaves were flash-frozen in liquid nitrogen and stored at −80 °C. To each sample, 300 μL extraction solvent composed of methanol, chloroform and formic acid (20:10:1, *v*/*v*/*v*) was used. The mixture was then vigorously shaken (using a paint shaker) for 5 min. Briefly, 150 μL of 0.2 M phosphoric acid, 1 M potassium chloride was added, and samples were centrifuged at 13,000× *g* at room temperature for 1 min. Lipids were extracted and followed by separation using two-dimensional thin-layer chromatography (TLC) on silica gel plates (pre-coated silica gel plates, Merck 5626) according to Xu and Siegenthaler [43]. The first developing solvent was acetone/toluene/H_2_O (91:30:8, by volume) and the second was chloroform/methanol/25% NH_3_/H_2_O (65:35:3:2, by volume). The plates were shortly air-dried before being delicately sprayed with 0.01% primuline and viewed under ultraviolet radiation. The transesterification of individual lipids previously separated by TLC was performed with 5% H_2_SO_4_ in methanol at 85 °C for 1 h. A Hewlett-Packard 5890 gas chromatography system equipped with a hydrogen flame ionization detector and an FFAP capillary column (30 m; i.d. 0.53 mm) was used to separate the fatty acid methyl esters. The column was run isothermally at 190 °C, while the detector was kept at 230 °C. The internal standard was heptadecanoic acid provided by Sigma Aldrich Chemical Co. (St. Louis, MO, USA). All other used chemicals were purchased from Merck (Darmstadt, Germany) and were of analytical purity.

### 4.6. Antioxidant Enzyme Activities

For protein extraction, young fresh leaves were collected from 6- and 12-week-old maize plants. The leaves (1 g) were immediately frozen in liquid nitrogen, lyophilized, and homogenized in 2-mL of 0.1 mM potassium phosphate (pH 7.8). The samples were centrifuged for 15 min at 12,000× *g* (4 °C). The supernatants were collected into tubes and stored at −20 °C until needed for enzymatic activity assays. The activity of superoxide dismutase (SOD) was measured spectrophotometrically by determining the inhibition of blue diformazan formation in the presence of riboflavin/nitroblue tetrazolium (NBT) and light [44]. The modified assay solution was prepared with 1-mL of 50 mM sodium phosphate (pH 7.8), 0.1 mM EDTA, 0.3 mM riboflavin, and 30 L of leaf extract. After 5 min at room temperature, the solution was mixed with NBT to obtain a final concentration of 0.03 mM NBT. The reaction mixture was then illuminated for 3 min with a fluorescent light (75 W, 20 cm above the mixture) and absorbance was determined at 560 nm. The reaction mixture without extract was used to calculate the control rate. NBT absorption was insignificant. The activity of SOD is presented in min^−1^ mg^−1^ protein with one unit described as 50% inhibition of blue diformazan formation. Also, the activity of Peroxidase (POD) was determined by measuring the increase rate in absorbance at 470 nm with o-dianisidine as the substrate [45]. The assay solution was 1 mL of 0.01 M sodium phosphate (pH 6.0) containing 1.3 mM H_2_O_2_, 1 mM o-dianisidine and 5 µL of extract. The activity was expressed as ∆OD_470 nm_ min^−1^ mg^−1^ protein.

### 4.7. Photosynthetic Pigments

Fresh leaves from 12-week-old maize plants were sampled for photosynthetic pigments assessment [46,47]. The leaves were finely cut into small sections (~0.1 g) and ground to a powder in 80% acetone (10 mL) and then centrifuged for 5 min at 10,000 rpm. After collecting the supernatant, the procedure was repeated until the residue was colorless. The absorbance of the solution was recorded at 480, 645 nm, and 663 nm. 80% acetone was included as the blank solution. The photosynthetic pigments of leaves were determined by estimating the contents of chlorophylls (Equations (1)–(3)) and carotenoids content by (Equation (3)).

Chlorophyll a (mg g^−1^ FW) = (0.0127 * A_663_) − (0.00269 * A_645_) * V/W (1)

Chlorophyll b (mg g^−1^ FW) = (0.0229 * A_645_) − (0.00468 * A_663_) * V/W (2)

Carotenoids (mg g^−1^ FW) = [A_480_ + (0.114 * A_663_)−(0.638 – A_645_)] * V/W (3)

where A_663_, A_645_, A_480_ are absorbance at 663, 645, and 480 nm, respectively, and V is the total volume of sample solution and W is the sample weight.

### 4.8. Statistical Analysis

Data were analyzed by one-way analysis of variance (ANOVA) using Genstat statistical software (ver.12). Significant differences among treatments were computed by Duncan’s multiple range tests (*p* < 0.05).

## 5. Conclusions

Maize cultivated on alkaline soils suffer from nutrient deficiency and this situation may become worse if such soils are affected by salinization. The use of biochar or AMF alone in amending such soils can mitigate the negative impacts of nutrient deficiency and low levels of salinization but become inefficient under high saline concentrations. Our study demonstrates that the combined application of biochar and AMF can effectively alleviate the adverse effects of saline stress on plant growth by (a) improving soil fertility, (b) increasing antioxidant enzymes activities and (c) increasing the levels of unsaturated fatty acid. Nevertheless, biochar application in alkaline and saline soils has shown contrasting results in different studies and this study adds to the limited literature on this. It is suggested that (1) broader studies be conducted in the greenhouse with different plants and different alkaline soils to have a larger picture of the effect of this amendment on alkaline soils under saline stress, (2) researchers should perform small scale field experiments with actual alkaline-saline soils to compare the mechanisms of different amendments in improving soil fertility, and (3) researchers should evaluate the difference in plant-growth improvement mechanisms of these amendments between actual alkaline-saline soils and alkaline soils which are salinized in laboratory studies.

## Figures and Tables

**Table 1:** Fatty acid composition in lipids of the maize plant.

Treatment	Fatty Acids (mol %)								
	C14:0	C16:0	C16:1	C18:0	C18:1	C18:2	C18:3	C20:0	C22:0
S0	1.84 ± 0.138a	12.7 ± 0.013a	0.786 ± 0.035bcd	11.5 ± 0.611a	10.8 ± 0.017b	27.4 ± 0.141b	28.4 ± 0.107b	2.48 ± 0.103i	2.29 ± 0.049g
S1	1.75 ± 0.151a	12.1 ± 0.216a	0.701 ± 0.011cdef	11.2 ± 0.064a	11.0 ± 1.07b	26.9 ± 0.06b	28.1 ± 0.017b	3.09 ± 0.005g	3.02 ± 0.002e
S2	1.81 ± 0.053a	11.9 ± 0.63a	0.540 ± 0.059ghi	11.5 ± 0.236a	8.92 ± 0.016c	21.8 ± 0.948cd	28.5 ± 1.18b	3.79 ± 0.077de	3.85 ± 0.15c
S3	1.90 ± 0.073a	12.4 ± 0.199a	0.440 ± 0.033ij	11.0 ± 1.13a	7.18 ± 0.556d	20.3 ± 0.425e	28.3 ± 0.587b	3.95 ± 0.055c	4.3 ± 0.167b
BS0	1.93 ± 0.056a	11.5 ± 1.51a	0.840 ± 0.081bc	11.7 ± 1.03a	10.7 ± 0.022b	27.5 ± 0.578b	28.8 ± 1.71b	2.46 ± 0.005i	2.32 ± 0.006g
BS1	1.84 ± 0.188a	11.8 ± 2.18a	0.640 ± 0.076defgh	11.0 ± 1.0a	11.1 ± 1.03b	27.2 ± 0.647b	28.0 ± 1.07b	3.70 ± 0.154e	2.79 ± 0.056f
BS2	1.84 ± 0.286a	12.0 ± 2.81a	0.560 ± 0.044fghi	12.2 ± 1.1a	8.06 ± 1.001cd	22.2 ± 1.53c	28.9 ± 1.04b	4.63 ± 0.017b	3.54 ± 0.006d
BS3	1.78 ± 0.161a	11.7 ± 0.793a	0.510 ± 0.055hi	11.7 ± 0.519a	6.92 ± 1.70d	20.8 ±0.054de	28.0 ± 2.31b	5.66 ± 0.111a	5.61 ± 0.053a
AS0	1.83 ± 0.031a	12.6 ± 0.574a	0.710 ± 0.071cdef	11.0 ± 0.049a	10.8 ± 0.577b	28.1 ± 0.021b	28.8 ±0.941b	2.49 ± 0.053i	2.29 ± 0.037g
AS1	1.79 ± 0.172a	12.2 ± 1.61a	0.680 ± 0.108defg	11.1 ± 1.29a	10.8 ± 0.585b	27.4 ± 0.584b	28.7 ± 0.58b	2.84 ± 0.063h	2.62 ± 0.05f
AS2	1.74 ± 0.057a	12.0 ± 1.47a	0.630 ± 0.044efgh	11.5 ± 1.1a	8.88 ± 0.028c	22.0 ± 1.217cd	28.1 ± 0.988b	2.71 ± 0.0005h	2.78 ± 0.052f
AS3	1.69 ± 0.112a	12.3 ± 1.14a	0.330 ± 0.249j	11.7 ± 1.77a	6.72 ± 0.527d	20.7 ± 0.051de	28.3 ± 0.498b	2.79 ± 0.059h	2.67 ± 0.302f
ABS0	1.84 ± 0.063a	11.9 ± 1.41a	0.760 ± 0.039cde	11.9 ± 1.24a	10.6 ± 0.06b	27.9 ± 0.554b	28.2 ± 1.00b	2.42 ± 0.025i	2.26 ± 0.051g
ABS1	1.90 ± 0.074a	12.4 ± 0.557a	0.920 ± 0.03b	12.0 ± 1.77a	10.3 ± 0.052b	27.9 ± 0.101b	28.6 ± 1.01b	3.53 ± 0.025f	2.72 ± 0.296f
ABS2	1.94 ± 0.042a	12.2 ± 0.641a	1.16 ± 0.001a	11.9 ± 1.24a	14.4 ± 1.16a	33.9 ± 1.52a	28.9 ± 0.973b	3.91 ± 0.051cd	3.043 ± 0.058e
ABS3	1.91 ± 0.184a	12.1 ± 1.15a	1.15 ± 0.01a	11.2 ± 1.02a	14.5 ± 0.579a	33.5 ± 0.57a	31.7 ± 1.53a	4.67 ± 0.194b	3.56 ± 0.034d

Data are presented as the means± standard deviation of three replicates and mean values followed by different letters are significantly different at *p* < 0.05 according to Duncan multiple range test.

**Table 2:** Treatment arrangement.

Code	Description
S0	0 mM NaCl without soil amendment
S1	50 mM NaCl without soil amendment
S2	100 mM NaCl without soil amendment
S3	150 mM NaCl without soil amendment
BS0	biochar without NaCl and AMF
BS1	biochar and 50 mM NaCl without AMF
BS2	biochar and 100 mM NaCl without AMF
BS3	biochar and 150 mM NaCl without AMF
AS0	AMF inoculation without NaCl and biochar
AS1	AMF inoculation with 50 mM NaCl but without biochar
AS2	AMF inoculation with 100 mM NaCl but without biochar
AS3	AMF inoculation with 150 mM NaCl but without biochar
ABS0	combined AMF inoculation and biochar but without NaCl
ABS1	combined AMF inoculation, biochar and 50 mM NaCl
ABS2	combined AMF inoculation, biochar and 100 mM NaCl
ABS3	combined AMF inoculation, biochar and 150 mM NaCl

## Data Availability

Not applicable.
